# Cocirculation of Four Infectious Bronchitis Virus Lineages in Broiler Chickens in the Eastern Region of Saudi Arabia from 2012 to 2014

**DOI:** 10.1155/2020/6037893

**Published:** 2020-03-31

**Authors:** Abdullah I. A. Al-Mubarak, Anwar A. G. Al-Kubati

**Affiliations:** ^1^Department of Microbiology, College of Veterinary Medicine, King Faisal University, Hofuf, Saudi Arabia; ^2^Department of Botany and Microbiology, College of Sciences, King Saud University, Saudi Arabia

## Abstract

Avian infectious bronchitis virus (IBV) is an evolving and dynamic virus that causes major economic losses for the poultry industry worldwide. Continuous evolution and emergence of new variants of this virus are the major challenges for controlling the disease with routine vaccination. Successful vaccination usually requires the use of a homologous vaccine, which in turn necessitates continuous investigation of the circulating strains. Herein, we performed a reverse transcriptase-polymerase chain reaction- (RT-PCR-) based investigation in broiler chicken flocks of the Eastern Region of Saudi Arabia. IBV was detected in 36.5% of the tested flocks (42 out of 115) from January 2012 to March 2014. Direct sequencing of hypervariable region-3 (HVR-3) of the Spike (S)-1 gene was performed, followed by phylogenetic analysis to determine the circulating IBV genotypes. Four lineages appear to coexist in this region, including the GI-13 or 4/91 IBV (31%), GI-16 or CK/CH/LDL/97I IBV (28.6%), GI-1 or Mass IBV (19%), and GI-23 or Middle East IBV (21.4%). The latter lineage include two subgroups: IS/720/99 IBV (16.7%) and IS/Variant2/98 IBV (4.7%). Some of the detections made in the 4/91 and Mass lineages are expected to belong to the vaccine strains. Lineages without a homologous vaccine in use (CK/CH/LDL/97I and Middle East) represent 50% of the isolates recovered in this study. Based on identity with the vaccine sequences, field observations, and frequent detection, these two lineages appear to be out of coverage of the IBV vaccines used in Saudi Arabia. This is the first time to identify Middle East lineage (IS/720/99 IBV and IS/Variant2/98 IBV) in the Eastern Region of Saudi Arabia.

## 1. Introduction

Infectious bronchitis (IB) is an acute highly contagious respiratory disease of chickens (*Gallus gallus domesticus*) that is present in virtually all regions with an intensive poultry industry. In addition to respiratory involvement, IB may also affect the urogenital and/or alimentary tracts. IB causes significant economic losses by adversely affecting weight gain and the quality and quantity of produced eggs as well as predisposing birds to secondary infections [1].

The causative agent, infectious bronchitis virus (IBV), belongs to group III *Coronaviruses* of family Coronaviridae. IBV is an enveloped virus with a single-stranded positive-sense RNA genome of approximately 27.6 kb. Four structural proteins (spike (S), envelope (E), membrane (M), and nucleocapsid (N)) were reported to be encoded by the IBV genome [2]. The glycosylated S protein is posttranslationally cleaved by cellular proteases into S1, which forms the spike bulb, and S2, which anchors S1 to the virion membrane. The S1 and S2 subunits have been shown to mediate viral attachment and membrane fusion, respectively. Virus neutralizing epitopes are located in the first and third quarters of the S1 glycoprotein [2, 3]. Three hypervariable regions (HVR)-1, 2, and 3 were reported to be located in the S1 subunit between amino acids 38 to 67, 91 to 141, and 274 to 387, respectively [4]. Genetic variability of these HVRs due to mutations and/or recombination was incriminated in the continuous emergence of new IBV serotypes/genotypes [5]. In this regard, substitution of 10 to 15 amino acids (2-3%) in the S1 subunit has been shown to result in generation of a new serotype [6].

Large numbers of IBV serotypes have been reported worldwide, some of which remain restricted to particular geographic areas, whereas others tend to spread over the majority of the world [5, 7]. Vaccination is the primary measure used to control IBV infection, but caution must be taken when choosing a vaccine strain(s) that should be homologous with the circulating IBV serotype(s). Vaccination-challenge studies have shown that protection by heterologous vaccines ranges between very poor to moderate depending on the criteria used. Poor cross protection may be induced by as little as a 5% difference in the S1 amino acid sequence, while most IBV serotypes differ from each other by 20–25% and up to 50% [6]. Accordingly, S1 nucleotide sequence similarity and genotyping approaches have been used successfully to predict cross-protection and circulating serotypes, with some exceptions [8, 9]. Recently, IBV was genotyped based on S1 sequences into 6 major genotypes (Genotype I (GI) to GVI] containing 32 lineages [10].

Few reports have evaluated IBV infection in Saudi Arabia. IBV infection in this region extends back to 1984 when Zwaagstra and others reported an IBV isolate without specifying its serotype [11]. The 4/91 IBV serotype (GI-13 lineage) was detected in Saudi Arabia in 2000 [12]. Vaccine strains belonging to this serotype and the Mass serotype (GI-1 lineage), such as H120 and Ma5, are in use to control IB occurrence; however, IB still occurs due to the CH/CK/LDL/97I (GI-16) lineage, the Middle East (GI-23) lineage (IS/720/99 and IS/Variant2/98), and D274 (GI-12) lineage [13, 14]. Strains similar to those circulating in Egypt, India, China, and Italy have also been reported in the Eastern Region of Saudi Arabia [15]. In the present study, we report the IBV lineages circulating in 115 broiler chicken flocks in the Eastern Region of Saudi Arabia.

## 2. Materials and Methods

### 2.1. Ethical Approval

All experimental procedures and management conditions used in this study were approved by the Ethics Committee at King Faisal University, Saudi Arabia.

### 2.2. Sampling and Nucleic Acid Extraction

The study was conducted between January 2012 and March 2014. Samples were collected from 115 broiler chicken flocks in the Eastern Region (Al-Hassa and Dammam) of Saudi Arabia by visiting broilers farms, broilers slaughterhouse, and the poultry clinic at the Veterinary Teaching Hospital of King Faisal University. Tissue samples, such as trachea, lung, spleen, kidney, and bursa of Fabricius, were collected from birds showing signs of respiratory illness. Although not always successful, attempts were made to collect various organs and a complete history from the sampled flocks. For each flock, each organ was pooled together from all sampled birds. Tissue samples were collected in 10 volumes of phosphate-buffered saline (PBS) containing gentamicin and nystatin (50 *μ*g of each/ml). The samples were stored at −80°C until being homogenized. Homogenization was performed using the Omni International Ceramic bead kit and the BioSpecMini-Beadbeater-16 instrument. For each organ, viral nucleic acids were extracted from homogenized tissues with the IQEasy Plus Viral DNA/RNA Extraction Kit (Cat #17153, iNtRON Biotechnology, South Korea) according to the manufacturer's instructions. The extracted nucleic acids were stored at −80°C until being tested by RT-PCR. The isolates were named according to the Cavanagh nomenclature system [16] that includes the country/sample name/sampling year.

### 2.3. Synthesis of cDNA

First-strand cDNA synthesis was performed using a Reverse Transcription System (Cat #A3500, Promega, USA) according to the manufacturer's instructions. The reaction was performed in a final volume of 20 *μ*l containing the following ingredients: 5 mM MgCl_2_, 1x RT buffer, 1 mM of each dNTP, 1 U/*μ*l of Recombinant RNasin® Ribonuclease Inhibitor, 0.75 U/*μ*l of AMV reverse transcriptase, 25 ng/*μ*l of Random Primers, and 50 ng/*μ*l of total RNA. The reaction mixture was incubated at 42°C for 45 minutes, followed by heat inactivation of the enzyme at 95°C for 5 minutes and 4°C for 5 minutes. The cDNA was diluted with nuclease-free water to a final volume of 50 *μ*l and stored at −80°C until being tested by PCR.

### 2.4. N Gene PCR

For diagnostic purposes, a nested PCR targeting the nucleocapsid (N) gene of IBV was carried out. The reaction was performed using the previously published primers N784, N1145, N791, and N1129 [17], which were obtained from Integrated DNA Technologies (IDT, Coralville, IA, USA) and presented in Table 1. The nested PCR reactions were performed using the Go Taq® Green Master Mix (Cat #M7122, Promega) in a final volume of 25 *μ*l containing the primers at a final concentration of 0.8 *μ*M with 2 *μ*l of the template cDNA. The Bio-Rad T-100 Thermal Cycler was used to perform an amplification reaction that consisted of 94°C for 5 minutes and 35 cycles of 94°C for 45 seconds, 60°C for 1 minute, and 72°C for 2 minutes, followed by a final extension step of 72°C for 7 minutes. A similar thermal profile was used for the second round of the nested PCR except that the annealing step was performed at 53°C for 1 minute and the extension step lasted for 1 minute and 10 minutes for the cyclic and final extension steps, respectively. The second-round PCR products were separated on a 1.5% agarose gel in Tris-Borate-EDTA (TBE) buffer. Red Safe (Cat #21141, iNtRON Biotechnology) was used as a DNA stain. Positive samples were subjected to the S1 gene PCR.

### 2.5. S1 Gene PCR

For sequencing purposes, another nested PCR targeting HVR-3 of the IBV-S1 gene was performed. We used previously published primers, including SX1, SX2, SX3, and SX4 [18], which were obtained from Integrated DNA Technologies (IDT, Coralville, IA) and presented in Table 1. A PCR was performed using i-StarMAX II (Cat #25174, iNtRON Biotechnology) in a final volume of 50 *μ*l. One microliter (final concentration 0.2 *μ*M) of each primer, 2 *μ*l of template cDNA, and 21 *μ*l of nuclease-free water were added to 25 *μ*l of the master mix. Thereafter, the reactions were treated in a similar manner as the second round of the N gene PCR except that the annealing temperature was 55°C for 1 minute for both rounds of the nested PCR. The second-round PCR product was separated on a 1.5% low melting point agarose gel in TBE buffer. The targeted bands were excised, and the amplicons were purified from the agarose gel using the Wizard SV Gel and PCR Cleanup System (Cat #A1460, Promega) according to the manufacturer's instructions. Purified amplicons were sequenced by Macrogen Sequencing Service (South Korea).

### 2.6. Isolation

The isolation protocol described by Gelb and Jackwood [19] was performed to propagate the virus in embryonated Specific Pathogen Free (SPF) eggs (Nile SPF, Egypt). Isolation was attempted from samples (Table 2) that gave positive results in the N gene PCR but turned either negative or weakly positive in the S1 gene PCR. Before inoculation, homogenized tissue samples were centrifuged at 1000 ×g for 10 minutes. The centrifugation-derived supernatant was passed through a 0.2 *μ*m sterile nylon syringe filter (Thermo Scientific, Nalgene®, Cat #195-2520, USA). A 200 *μ*l volume of filtrated liquid was inoculated into the allantoic cavity in 10-day-old embryonated eggs. Forty-eight hours postinoculation, inoculated eggs were placed at 4°C overnight. Allantoic fluids (AFs) were collected and used as inocula, at a rate of 100 *μ*l/egg, for additional two passages. AFs collected from the third passage were retested by RT-PCR.

### 2.7. Sequence Analysis

**Figure 1:**
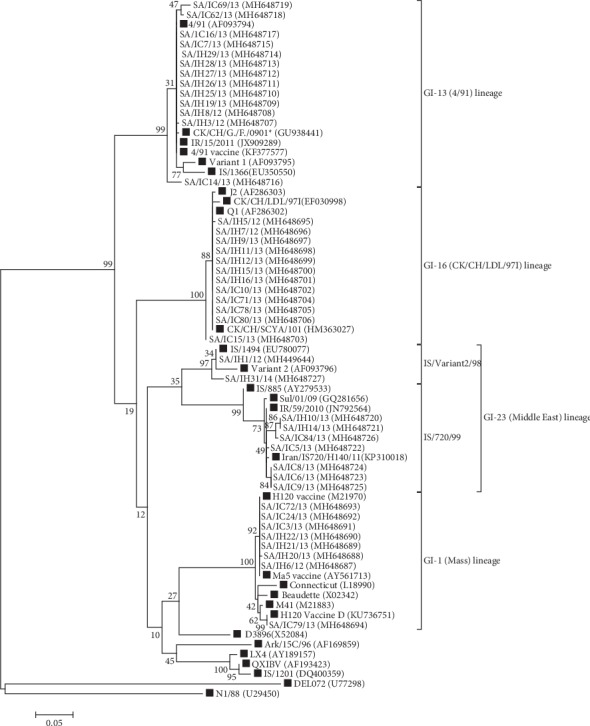
Phylogenetic tree showing the relationship between the detected IBV isolates and the reference sequences over the sequenced region of the S1 gene. Reference sequences are tagged with a black square. The GenBank accession numbers are shown in brackets. ^*∗*^CK/CH/Guangdong/Fengmulang/0901 (GB#GU938441).

The sequence analysis was performed using the Molecular Evolutionary Genetic Analysis (MEGA) version X software [20]. Sequences were aligned using the Clustal W method. The aligned sequences were trimmed to a length of 327 nt. (from nt. 718 to 1044 based on the 793/B S1 gene sequence (GB#Z83979)). A phylogenetic tree was constructed using the maximum likelihood method with bootstrap values of 1000 replicates. Twenty-nine IBV reference sequences were included (Figure 1). The overall mean distance was used as an indicator of sequence divergence for the Saudi isolates and was shown as the number of different bases per sequence from averaged overall sequence pairs. Sequence identity was calculated using the BioEdit version 7.1.7 software [21]. A BLAST search was performed for all sequences to determine the most related sequence in GenBank.

### 2.8. Vaccination History

Vaccination histories for the flocks in which IBV was detected are presented in Table 2. Regarding the 13 flocks in which 4/91 IBV was detected, four flocks received only the 4/91 vaccine, one flock received both the Mass and 4/91 vaccines, and four flocks received only the Mass vaccine twice. Histories were not available for the other four flocks. For the 12 flocks in which the CK/CH/LDL/97I IBV was detected, eight flocks received the Mass vaccine either once (*n* = 5) or twice (*n* = 3), one flock received the 4/91 vaccine once, and histories were not available for the additional three flocks. In the 8 flocks that were positive for the Mass IBV, five flocks received the Mass vaccine, and histories were not available for the other three flocks. Concerning the seven flocks positive for the IS/720/99 IBV, 2 flocks received the H120 vaccine and one received the 4/91 vaccine; histories were not available for the other 4 flocks. Finally, the two flocks in which IS/Variant2/98 was detected had received the Mass vaccine.

### 2.9. Differentiation between Field and Vaccine Viruses

The methods used in this study could not definitely determine the fraction contributed by the vaccine viruses; however, removal of sequences with 100% nucleotide identity to the reference vaccine sequences was used only as a guide, as previously reported by [14, 18, 22]. Comparisons were performed with the two Mass vaccines (H120 and Ma5) and with the 4/91 vaccine.

### 2.10. GenBank Accession Numbers

The sequences of the 42 IBV isolates from the present study have been deposited in the GenBank database with accession numbers MH648687 to MH648727 and MH449644, as shown in Figure 1 and Table 2.

## 3. Results

### 3.1. Detection Rate and Genotyping

During the study period, tissue samples were collected from 115 broiler flocks in the Eastern Region of Saudi Arabia. Samples from forty-two flocks (36.52%) were positive for IBV in the N and S1 genes RT-PCRs (Table 2) either directly or after isolation in SPF eggs. Isolation were performed for 7 samples including SA/IH1/12, SA/IH5/12, SA/IH11/13, SA/IH19/13, SA/IH21/13, SA/IC8/13, and SA/IC15/13. Phylogenetic analysis based on partial S1 gene sequences of these forty-two Saudi isolates and twenty-nine reference sequences showed segregation of these isolates into four lineages (Figure 1 and Supplementary [Supplementary-material supplementary-material-1]) including GI-13 (4/91), GI-16 (CK/CH/LDL/97I), GI-1 (Mass), and GI-23 (Middle East) that include two subdivisions: IS/720/99 and IS/Variant2/98 IBVs.

### 3.2. GI-13 or 4/91 Lineage

The first cluster included thirteen sequences that were grouped with 4/91, IS/Variant1, IS/1366, and other reference sequences. A BLAST search for 4/91-like sequences showed that the 4/91 strain (GB#AF093794) was the most similar sequence, with identities ranging between 96.9% and 100% over the sequenced region of the S1 gene. Sequences of this lineage showed an overall mean distance of 3.000 ± 0.720 nucleotides and an average nucleotide identity of 99.5% with the 4/91 vaccine (Table 3).

### 3.3. GI-16 or CK/CH/LDL/97I Lineage

The second lineage included twelve sequences that clustered around the CK/CH/LDL/97I, CK/CH/SCYA/101, Q1, and J2 reference sequences. A BLAST search for CK/CH/LDL/97I-like sequences showed that Chinese Q1 was the most similar sequence, with nucleotide identities ranging between 99.0% and 100% over the sequenced part of the S1 gene. The overall mean distance was 0.667 ± 0.316 nucleotides. The average nucleotide identity of these sequences with the IBV vaccines was 79.5% with the H120 and Ma5 vaccines and 81.7% with the 4/91 vaccine (Table 3).

### 3.4. GI-1 or Mass Lineage

Eight Saudi sequences were included in this lineage together with the Mass serotype strains, including the H120, Ma5, M41, Beaudette, and Connecticut reference sequences. A BLAST search for Mass-like sequences revealed H120 as the most similar sequence, with identities ranging between 97.8% and 100% over the sequenced part of the S1 gene. The overall mean distance of this group was 2.000 ± 0.674 nucleotides. The presented sequences showed a 99.7% average nucleotide identity with the H120 and Ma5 vaccines (Table 3).

### 3.5. GI-23 or Middle East Lineage

The fourth lineage included nine Saudi isolates and further subdivided into two subgroups: IS/720/00 IBV and IS/Variant2/98 IBV. IS/720/99 IBV included seven Saudi isolates that were assembled around the Middle East Sul/01/09, IR/59/2010, IS/885/00, and IS/720/99 reference sequences. A BLAST search for IS/720/99-like sequences revealed that IR/IS720/H140/11 was the most related sequence, with identities ranging between 97.8% and 99.3% over the sequenced region of the S1 gene. The overall mean distance for these sequences was 4.952 ± 1.463 nucleotides, and their nucleotide identities were on average 81.9% with both the Mass vaccines and the 4/91 vaccine (Table 3). In the other subgroup, IS/Variant2/98 IBV, only two Saudi sequences were located with the IS/Variant2/98 and IS/1494/06 reference sequences. A BLAST search for the IS/Variant2/98-like sequences showed that IS/1494/06 was the most similar sequence, with identities ranging between 97.8% and 100% over the sequenced part of the S1 gene. The overall mean distance was 7.000 ± 2.552 nucleotides. The average nucleotide identity was 82.2% with the Mass vaccines and 83.0% with the 4/91 vaccine (Table 3).

### 3.6. Field and Vaccine Viruses

At least two types of IBV vaccines (Mass and 4/91) are in use in Saudi Arabia. Hence, isolates in these two lineages could belong to vaccine viruses. We compared the partial S1-gene sequences of isolates in these lineages with those of the relevant vaccines. Nine out of the 13 sequences in 4/91 lineage and 6 out of the 8 sequences in Mass lineage showed complete nucleotide identities with corresponding vaccine sequences over the sequenced part of S1 gene (Table 2).

## 4. Discussion

The present investigation documents the IBV lineages circulating in broiler chicken flocks of the Eastern Region of Saudi Arabia during the period from January 2012 to March 2014. For IBV detection, we used a validated and highly sensitive nested PCR that targeted the conserved part of the N gene and was used frequently in other studies [13, 17, 23, 24].

In the current work, IBV was detected in 36.5% of the tested flocks. A slightly higher detection rate (42.7%) was reported in Riyadh between October 2009 and May 2010 [25]. Caution should be taken in interpreting these results because samples were collected only from flocks showing signs of respiratory illness. Similar investigations revealed detection rates as high as 74% in south Iraq [26], 64% in Egypt [27], 60% in Jordan [28], 59% in western Europe [18], 52% in Iran [29], 37.5% in eastern Iran [30], 34% in Russia [22], and 32% in middle-south Iraq [31]. Testing flocks regardless of their clinical picture are expected to reveal lower detection rates as previously reported by Chen and others [32]. Furthermore, vaccine isolates represent a fraction of this percentage.

Comparison of sequences of the present Saudi isolates with those of the IBV vaccines revealed that six and nine isolates from the Mass and 4/91 lineages, respectively, had vaccine-identical sequences (Table 2). Comparable vaccine detection rates were reported in other RT-PCR-based investigations, where vaccines represented approximately half or more of the total detections made in the vaccines lineages [14, 18, 22]. If the vaccine-identical sequences were removed, the CK/CH/LDL/97I lineage would become the dominant lineage in the Eastern Region of Saudi Arabia.

In Saudi Arabia, the CK/CH/LDL/97I (GI-16) lineage was first reported in Riyadh in 2009–2010 [25]. Then, this strain was reported in 2011 in Saudi Arabia, Jordan, and Iraq by Ababneh and colleagues [13]. In the present study, the CK/CH/LDL/97I lineage was detected at a rate of 28.6% (12/42) between January 2012 and March 2014 in the Eastern Region of Saudi Arabia. A detection rate of 17% for this lineage was reported from Saudi Arabia during an overlapping period from 2009 to 2014 [14]. Conversely, this strain was either absent or had a low prevalence rate in surrounding countries because it was not detected in studies in Egypt, Lebanon, Jordan, Kuwait, the United Arab Emirate (UAE) and Oman [14, 33], Iraq [26, 31], and Iran [29, 30].

Our data showed that 8 of the 12 flocks in which the CK/CH/LDL/97I lineage was detected received only the Mass vaccine and that one received only the 4/91 vaccine; histories were not available for the remaining three flocks. This finding was not surprising because the original CK/CH/LDL/97I strain was isolated from an H120-vaccinated flock. Likewise, a vaccination-challenge experiment showed that heterologous vaccines did not provide sufficient protection against CK/CH/LDL/97I IBV. In contrast, complete protection was provided by a homologous vaccine derived from the same type and attenuated by passaging in embryonated hen eggs [34]. In Saudi Arabia, the high prevalence of this lineage in broiler chickens appears to be facilitated by an immunity gap created by use of only heterologous vaccines. A similar scenario was suggested for spread of the IT-02 IBV strain in Spanish 4/91-vaccinated flocks [9].

The 4/91 (G1-13) lineage was first reported in Saudi Arabia in 2000 [12]. In the present investigation, the 4/91 lineage was detected at a rate of 31% in the Eastern Region of Saudi Arabia between 2012 and 2014. Similarly, it was the most prevalent lineage with a detection rate of 43% in Saudi Arabia during the period from 2009 to 2014 [14] and had reported detection rates of 40% and 50% in Iraq [26, 31], 64% and 81% in Oman, and 85% in the UAE [14, 33]. The 4/91 lineage was also the second most prevalent lineage in Iran (19% and 27%), Egypt (17%), and Lebanon (13%) but was undetectable in Jordan and Kuwait [14, 29, 30].

In the current study, approximately two-thirds of the 4/91 lineage isolates (9 out of 13) showed 100% sequence identity with the 4/91 vaccine virus. These isolates most likely belong to the vaccine virus. This speculation is supported by the history of recent use of the 4/91 vaccine in 4 out of these 9 flocks. Conversely, four of these nine flocks received only the Mass vaccine, and the vaccination history was not available for the ninth flock. Keeping in mind that approximately 70% of the 4/91-like sequences were vaccine-identical, and this lineage contains some isolates that displayed relatively high sequence divergence (Table 3). Heavy vaccine application may contribute to such divergence, as previously suggested by Lee and Jackwood [35]. In contrast, the CK/CH/LDL/97I lineage showed the lowest divergence, which could further support our justification of the low immune pressure exerted by the used vaccines on CK/CH/LDL/97I lineage viruses.

The Mass (GI-1) lineage was detected at a rate of 19% in the present study. A similar rate (17%) was reported by Ganapathy and colleagues (2015) in Saudi Arabia during the 2009–2014 period. A relatively lower prevalence was reported in surrounding countries, including Egypt (8%), Lebanon (4%), the UAE (4%), Iraq (6%), Oman (6% and 3%), and Iran (3%), and it was undetectable in Jordan and Kuwait [14, 26, 29, 33].

Out of the eight isolates recovered for the Mass lineage, six showed vaccine-identical sequences and were likely to belong to the Mass vaccine strain. In favor of this opinion, four out of these six vaccine-identical isolates were obtained from flocks that received the Mass vaccines; histories were not available for the remaining two flocks. On the contrary, two out of the eight Mass isolates showed incomplete identity with Mass vaccines (SA/IH20/13 and SA/IC79/13). SA/IH20/13 showed 99.6% nucleotide identity with the Mass vaccine sequence and was recovered from a Mass-vaccinated flock (Table 2). Similarly, SA/IH3/12 (4/91-like isolate) showed 99.6% nucleotide identity with the 4/91 vaccine sequence and was recovered from a 4/91-vaccinated flock. Whether these isolates belong to field viruses or to mutant vaccine viruses is not clear. Nucleotide mutations and subsequent amino acid substitutions may affect vaccine protectivity or even lead to vaccine reversion. An escape mutant of the Mass M41 strain was reported to occur due to a single nucleotide mutation at position 134, leading to an amino acid change at position 45 of HVR-1 of the S1 gene [36].

In the current study, the IS/720/99 IBV of the Middle East (GI-23) lineage was detected in 7 flocks (16.7%) in the Eastern Region of Saudi Arabia during the period from 2012 to 2014. This subdivision was first reported in Saudi Arabia in 2010 by Ganapathy and others and up to 2014 was detected in 11% of IBV-positive samples [14]. In the present study, all seven isolates were recovered in 2013 (Table 2). Similarly, most of the detections by Ganapathy and colleagues were made in 2013, whereas this virus was almost undetectable in 2011 and 2012 [14]. This difference in the detection rate may reflect the time of introduction and ongoing spread of this genotype inside the country. The IS/720/99 IBV was shown to have a low prevalence in Oman (3%) and was not detected in Jordan, Lebanon, Kuwait, the UAE, Iraq, and Iran. In contrast, IS/720/99 IBV was the most prevalent (71%) in Egypt [14, 26, 29–31, 33].

Vaccination histories were available for 3 of the 7 flocks in which the IS/720/99 IBV was detected. Two flocks received the Mass vaccine, and one flock received the 4/91 vaccine. A vaccination-challenge experiment showed that the H120 vaccine provided little protection against IS/720/99 [37]. Over the sequenced region in the present study, genetic relatedness between the IS/720/99-like isolates and the Ma5 or 4/91 vaccines was not better than that with H120. Therefore, we can rationally expect poor cross-protection with these heterologous vaccines.

The other subdivision of the Middle East (GI-23) lineage detected in this study was the IS/Variant2/98 IBV. Our data showed that the IS/Variant2/98 IBV had the lowest prevalence (4.7%) in Eastern Region of Saudi Arabia. From 2009 to 2014, a prevalence of 11% was reported in Saudi Arabia and 4% in Egypt, and the strain was not detected in the UAE [14]. Similarly, variable prevalence rates of 21% and 3% were reported in Oman during overlapping periods from 2009 to 2014 and during 2012, respectively [14, 33]. In contrast, this strain was reported to be the most prevalent in the neighboring north countries, including prevalence rates of 100% in Jordan and Kuwait; 82% in Lebanon; 67%, 70%, and 75% in Iran; and 47% in Iraq [14, 29–31, 38].

The two isolates within the IS/Variant2/98 subdivision reported in this study were recovered from two Mass-vaccinated broiler flocks. Based on reported field observations of H120-vaccinated flocks [39] and sequence similarity of the detected isolates with available vaccines (Mass and 4/91), which showed ≤83% sequence identity (Table 3), these vaccines seem unlikely to be able to provide protection against IS/variant2-like strains.

As stated above, considerable variation existed between the detection rates reported from different countries and even from the same countries, for example, the difference in the detection rates of 4/91 lineage in Saudi Arabia reported by this study and Ganapathy and others (2015) and the above mentioned difference in the detection rates of IS/Variant2/98 IBV in Oman. Likewise, other lineages were reported in surrounding countries but not in Saudi Arabia or vice versa; for example, the QX (or GI-19) lineage was detected at rates of 7% and 8% in Iran and 9% in Iraq [29–31]. Similarly, Ganapathy and colleagues (2015) detected the D274 (or GI-12) lineage at a frequency of once out of 236 samples during the period from 2009 to 2014, whereas we did not detect this lineage at the Eastern Region of Saudi Arabia during the period from 2012 to 2014. Similarly, the CK/CH/LDL/97I lineage was prevalent in Saudi Arabia but not in surrounding countries. Several factors may contribute to this inconsistency, including but not limited to the following: (1) differences in the sampled population, such as those that arise from sampling different birds (broilers, layers, breeders etc.); (2) serotype(s) of the used vaccines, frequency of their application, and the protection they provide against the circulating genotypes; (3) the effect of migratory birds and localization of the sampled flocks on the migration route [40, 41]; (4) applied biosecurity measures; and (5) exchange of poultry products between countries and regions [13]. Additionally, testing different tissues would also contribute to variation in the detection rates. IBV vaccine and field viruses, or their genome/antigen, were detected in variety of tissues [42–44] and reported to persist in some viscera, particularly cecal tonsils [18, 45–48]. Consequently, testing the latter tissue would increase the detection rate on the one hand and increase the fraction contributed by vaccine viruses, on the other hand, as previously speculated by Callison and others [49].

In the present study, IBV genotyping was performed by direct sequencing of amplicon produced by nested RT-PCR that targets the HVR-3 of the S1 gene [18]. Since being introduced, this method has been frequently used for IBV genotyping [14, 29, 50–52]. Advantages and disadvantages of full and partial sequencing of IBV-S1 gene was previously reported [53]. According to the phylogenetic analysis of the partial (HVR-3) S1 gene sequences published by Valastro et al. [10], all of the lineages involved in the present study (G1-1, -13, -16, and -23) formed distinct and well-supported clusters (Shimodaira–Hasegawa-like test support values of ≥0.93). Indeed, Valastro et al. specify 8 lineages (GI-5, -7, -10, -18, -22, -24, -25, and -27) that loss their unity in phylogeny when using the partial 342 nt. encoding the HVR3 [10]. However, the main limitation of the current work is the partial sequence of the S1 gene that was used for phylogenetic analysis. Full S1 gene sequence would provide more accurate analysis especially for differentiation between field and vaccine viruses.

In conclusion, our data show that IB is a prevalent disease in broiler chicken flocks of the Eastern Region of Saudi Arabia. Four IBV lineages were found to cocirculate in this region. Half of the detected isolates belong to two of these lineages (CK/CH/LDL/97I and Middle East) against which no homologues vaccines are available, and these lineages are expected to keep causing IBV outbreaks and economic losses in the absence of such vaccines. Further studies to elucidate the serotypic, pathotypic, and protectotypic properties of the endemic types are required. Continuous monitoring of the IBV types circulating in Saudi Arabia as well as continuous update of the vaccination strategy seem to be necessary to control IBV infection.

## Figures and Tables

**Table 1:** Primers used for IBV detection and sequencing targeting the N and S1 genes, respectively.

Primer	Primer sequence 5′ to 3′	Position in targeted gene	Reference
N784	AATTTTGGTGATGACAAGATGA	763–785^A^	[17]
N1145	CATTGTTCCTCTCCTCATCTG	1145–1165^A^	
N791	GTGATGACAAGATGAATGAGGA	770–791^A^	
N1129	CAGCTGAGGTCAATGCTTTATC	1129–1150^A^	

SX1	CACCTAGAGGTTTGT/CTA/TGCAT	677–698^B^	[18]
SX2	TCCACCTCTATAAACACC C/TTT	1148–1168^B^	
SX3	TAATACTGG C/T AATTTTTCAGA	705–725^B^	
SX4	AATACAGATTGCTTACAACCACC	1075–1097^B^	

^A^Targeted sequence in the N gene of IBV according to IBV strain Beaudette, GB# M95169. ^B^Targeted sequence in the IBV-S1 gene according to IBV strain 793/B, GB# Z83979.

**Table 2:** Detected IBV lineages in the Eastern Region of Saudi Arabia from 2012 to 2014 and associated sample data.

Isolate	Year of isolation	Tissue origin	Governorate	IBV vaccine age at vaccination	Detected IBV lineage/vaccine	GenBank accession #
SA/IH1/12^A^	2012	Trachea	Al-Hassa	CHB^*∗*^-1 day, H120-10 day	GI-23, IS/Variant2/98	MH449644
SA/IH3/12	2012	Trachea	Dammam	H120-1 day, 4/91-14 day	GI-13, 4/91	MH648707
SA/IH5/12^A^	2012	Trachea	Dammam	CHB-1 day	GI-16, CK/CH/LDL/97I	MH648695
SA/IH6/12	2012	Kidney	Al-Hassa	CHB-1 day, H120-12 day	GI-1, Mass vaccine-identical^B^	MH648687
SA/IH7/12	2012	Kidney	Al-Hassa	CHB-1 day, H120-10 day	GI-16, CK/CH/LDL/97I	MH648696
SA/IH8/12	2012	Kidney	Al-Hassa	CHB-1 day, H120-11 day	GI-13, 4/91 vaccine-identical^B^	MH648708
SA/IH9/13	2013	Lung	Al-Hassa	CHB-1 day, Ma5-10 day	GI-16, CK/CH/LDL/97I	MH648697
SA/IH10/13	2013	Kidney	Al-Hassa	H120-1 day	GI-23, IS/720/99	MH648720
SA/IH11/13^A^	2013	Kidney	Al-Hassa	H120-12 day	GI-16, CK/CH/LDL/97I	MH648698
SA/IH12/13	2013	Kidney	Al-Hassa	Ma5-1 day, Ma5-7 day	GI-16, CK/CH/LDL/97I	MH648699
SA/IH14/13	2013	Trachea	Dammam	H120-1 day	GI-23, IS/720/99	MH648721
SA/IH15/13	2013	Kidney	Al-Hassa	H120-1 day	GI-16, CK/CH/LDL/97I	MH648700
SA/IH16/13	2013	Kidney	Al-Hassa	H120-1 day	GI-16, CK/CH/LDL/97I	MH648701
SA/IH19/13^A^	2013	Trachea	Al-Hassa	CHB-1 day, H120-12 day	GI-13, 4/91 vaccine-identical	MH648709
SA/IH20/13	2013	Trachea	Al-Hassa	CHB-1 day, H120-10 day	GI-1, Mass	MH648688
SA/IH21/13^A^	2013	Trachea	Al-Hassa	CHB-1 day, H120-10 day	GI-1, Mass vaccine-identical	MH648689
SA/IH22/13	2013	Trachea	Al-Hassa	CHB-1 day, H120-10 day	GI-1, Mass vaccine-identical	MH648690
SA/IH25/13	2013	Trachea	Dammam	H120-1, day, Ma5-14 day	GI-13, 4/91 vaccine-identical	MH648710
SA/IH26/13	2013	Lung	Al-Hassa	4/91-1 day	GI-13, 4/91 vaccine-identical	MH648711
SA/IH27/13	2013	Trachea	Al-Hassa	4/91-1 day	GI-13, 4/91 vaccine-identical	MH648712
SA/IH28/13	2013	Trachea	Al-Hassa	4/91-1 day	GI-13, 4/91 vaccine-identical	MH648713
SA/IH29/13	2014	Trachea	Al-Hassa	4/91-1 day	GI-13, 4/91 vaccine-identical	MH648714
SA/IH31/14	2014	Trachea	Al-Hassa	H120-1 day	GI-23, IS/Variant2/98	MH648727
SA/IC3/13	2013	Trachea	Al-Hassa	H120-1 day, Ma5-14 day	GI-1, Mass vaccine-identical	MH648691
SA/IC5/13	2013	Trachea	Al-Hassa	NA	GI-23, IS/720/99	MH648722
SA/IC6/13	2013	Trachea	Al-Hassa	4/91-1 day	GI-23, IS/720/99	MH648723
SA/IC7/13	2013	Trachea	Al-Hassa	NA	GI-13, 4/91 vaccine-identical	MH648715
SA/IC8/13^A^	2013	Trachea	Al-Hassa	NA	GI-23, IS/720/99	MH648724
SA/IC9/13	2013	Trachea	Al-Hassa	NA	GI-23, IS/720/99	MH648725
SA/IC10/13	2013	Trachea	Al-Hassa	CHB-1 day	GI-16, CK/CH/LDL/97I	MH648702
SA/IC14/13	2013	Trachea	Al-Hassa	NA	GI-13, 4/91	MH648716
SA/1C15/13^A^	2013	Trachea	Al-Hassa	4/91-1 day	GI-16, CK/CH/LDL/97I	MH648703
SA/IC16/13	2013	Trachea	Al-Hassa	CHB-1 day, Ma5-10 day	GI-13, 4/91 vaccine-identical	MH648717
SA/IC24/13	2013	Trachea	Al-Hassa	NA	GI-1, Mass vaccine-identical	MH648692
SA/IC62/13	2013	Trachea	Al-Hassa	NA	GI-13, 4/91	MH648718
SA/IC69/13	2013	Trachea	Al-Hassa	NA	GI-13, 4/91	MH648719
SA/IC71/13	2013	Trachea	Al-Hassa	NA	GI-16, CK/CH/LDL/97I	MH648704
SA/IC72/13	2013	Trachea	Al-Hassa	NA	GI-1, Mass vaccine-identical	MH648693
SA/IC78/13	2013	Trachea	Al-Hassa	NA	GI-16, CK/CH/LDL/97I	MH648705
SA/IC79/13	2013	Trachea	Al-Hassa	NA	GI-1, Mass	MH648694
SA/IC80/13	2013	Trachea	Al-Hassa	NA	GI-16, CK/CH/LDL/97I	MH648706
SA/IC84/13	2013	Trachea	Al-Hassa	NA	GI-23, IS/720/99	MH648726

^*∗*^The Izovac CHB vaccine contains IBV Mass (H120 and BNF 28/86) and NDV clone. H120 (GB#M21970) showed complete nucleotide identity throughout the S1 gene with 28/86 strains (GB#AY846750). ^A^Samples for which isolation in embryonated SPF eggs were performed. ^B^Identities based on sequenced part of the S1 gene. ND: data were not available.

**Table 3:** The proportions of the detected lineages, range of identity with the most related sequence in GenBank, and relatedness with the used vaccines based on homology over the sequenced part of S1 gene.

Lineages [10]	GI-13 (4/91)	GI-16 (CK/CH/LDL/97I)	GI-1 (Mass)	GI-23 (IS/720/99)	GI-23 (IS/Variant2/98)
No. of isolates (%)	13 (31%)	12 (28.6%)	8 (19%)	7 (16.7%)	2 (4.7%)
Overall mean distance (nt.) ± SD	3.000 ± 0.720	0.667 ± 0.316	2.000 ± 0.674	4.952 ± 1.463	7.000 ± 2.552
BLAST most similar sequence, GB#	4/91 AF093794	Q1 AF286302	H120 M21970	IR/IS720/H140/11 KP310018	IS/1494/06 EU780077
Range of nt. identity with most similar sequence in GB (%)	96.9–100%	99.0–100%	97.8–100%	97.8–99.3%	97.8–100%
Average nt. identity with the 4–91 vaccine	99.50%	81.70%	HVA	81.90%	83.00%
Average nt. identity with the Mass vaccines (H120 and Ma5)	HVA	79.50%	99.70%	81.90%	82.20%

SD, standard deviation; nt., nucleotide; GB, GenBank; GB#, GenBank accession number; HVA, homologous vaccine is available.

## Data Availability

The epidemiological data used to support findings of this article are included within the article. The sequence data used to support findings of this article were deposited in GenBank under accession numbers shown in the article.

## Supplementary Materials

Supplementary Materials Supplementary Table 1: nucleotide identity matrix based on partial S1 gene sequences from seventeen IBV isolates and the most related reference sequences. The reference sequences are shown in bold font. Sequences with 100% identity are not shown, including the following: SA/IH9/13, SA/IH11/13, SA/IH12/13, SA/IH15/13, SA/IH16/13, SA/IC10/13, SA/IC71/13, SA/IC78/13, and SA/IC80/13 resembling SA/IH7/12; SA/IH19/13, SA/IH25/13, SA/IH26/13, SA/IH27/13, SA/IH28/13, SA/IH29/13, SA/IC7/13, and SA/IC16/13 resembling SA/IH8/12; SA/H21/13, SA/IH22/13, SA/IC3/13, SA/IC24/13, and SA/IC72/13 resembling SA/IH6/12; SA/H14/13 resembling SA/H10/13; and SA/C8/13 and SA/IC9/13 resembling SA/IC6/13.
