# Supplementary material for: Cocirculation of Four Infectious Bronchitis Virus Lineages in Broiler Chickens in the Eastern Region of Saudi Arabia from 2012 to 2014
Source: Vet Med Int. 2020 Mar 31;2020:6037893. doi: 10.1155/2020/6037893 (PMC7150681; doi:10.1155/2020/6037893)
Supplement: Supplementary Materials — Supplementary Table 1: nucleotide identity matrix based on partial S1 gene sequences from seventeen IBV isolates and the most related reference sequences. The reference sequences are shown in bold font. Sequences with 100% identity are not shown, including the following: SA/IH9/13, SA/IH11/13, SA/IH12/13, SA/IH15/13, SA/IH16/13, SA/IC10/13, SA/IC71/13, SA/IC78/13, and SA/IC80/13 resembling SA/IH7/12; SA/IH19/13, SA/IH25/13, SA/IH26/13, SA/IH27/13, SA/IH28/13, SA/IH29/13, SA/IC7/13, and SA/IC16/13 resembling SA/IH8/12; SA/H21/13, SA/IH22/13, SA/IC3/13, SA/IC24/13, and SA/IC72/13 resembling SA/IH6/12; SA/H14/13 resembling SA/H10/13; and SA/C8/13 and SA/IC9/13 resembling SA/IC6/13. [file 6037893.f1.pdf]

Supplementary Table 1: Nucleotide identity matrix based on partial S1 gene sequences from seventeen IBV isolates and the most related reference sequences. The reference sequences are shown in bold font. Sequences with 100% identity are not shown, including the following: SA/IH9/13, SA/IH11/13, SA/IH12/13, SA/IH15/13, SA/IH16/13, SA/IC10/13, SA/IC71/13, SA/IC78/13 and SA/IC80/13 resembling SA/IH7/12; SA/IH19/13, SA/IH25/13, SA/IH26/13, SA/IH27/13, SA/IH28/13, SA/IH29/13, SA/IC7/13 and SA/IC16/13 resembling SA/IH8/12; SA/H21/13, SA/IH22/13, SA/IC3/13, SA/IC24/13 and SA/IC72/13 resembling SA/IH6/12; SA/H14/13 resembling SA/H10/13; and SA/C8/13 and SA/IC9/13 resembling SA/IC6/13.

Seq->	**1 Q1 (AF286302)**	2 SA/IH5/12	3 SA/IH7/12	4 SA/IC15/13	**5 4/91 (AF093794)**	6 SA/IH3/12	7 SA/IH8/12	8 SA/IC14/13	9 SA/IC62/13	10 SA/IC69/13	**11 H120 vaccine (M21970**	12 SA/IH6/12	13 SA/IH20/13	14 SA/IC79/13	**15 Iran/IS720/H140/11 (KP310018)**	16 SA/IH10/13	17 SA/IC5/13	18 SA/IC6/13	19 SA/IC84/13	**20 IS/1494 (EU780077)**	21 SA/IH1/12	22 SA/IH31/14	Seq->
**1**																							**1**
2	99.6																						2
3	100	99.6																					3
4	99	98.7	99																				4
**5**	81.6	81.3	81.6	82.5																			**5**
6	81.3	81	81.3	82.2	99.6																		6
7	81.6	81.3	81.6	82.5	100	99.6																	7
8	81	80.7	81	81.9	96.9	96.6	96.9																8
9	81.9	81.6	81.9	82.8	99	98.7	99	96															9
10	82.2	81.9	82.2	83.1	98.1	97.8	98.1	95.7	98.4														10
**11**	79.5	79.2	79.5	79.8	77.3	77.3	77.3	77	78.2	77.9													**11**
12	79.5	79.2	79.5	79.8	77.3	77.3	77.3	77	78.2	77.9	100												12
13	79.5	79.2	79.5	79.8	77.3	77.3	77.3	77	78.2	77.9	99.6	99.6											13
14	78.5	78.2	78.5	78.8	77	76.7	77	76.7	77.9	77.6	97.8	97.8	97.5										14
**15**	80.4	80.7	80.4	81	82.2	81.9	82.2	81.3	81.9	82.2	82.2	82.2	81.9	81.3									**15**
16	79.5	79.5	79.5	80.1	81.3	81	81.3	81	81	81.3	81.3	81.3	81	80.4	97.8								16
17	80.1	80.4	80.1	80.7	81.9	81.6	81.9	81	81.6	81.9	81.6	81.6	81.3	80.7	99.3	97.8							17
18	80.7	81	80.7	81.3	82.2	81.9	82.2	81.3	81.9	82.2	82.2	82.2	81.9	81.3	99	97.5	99						18
19	80.1	80.1	80.1	80.7	82.2	81.9	82.2	81.3	81.9	82.2	82.2	82.2	81.9	81.3	98.1	99	98.1	97.8					19
**20**	83.1	82.8	83.1	83.7	83.1	83.1	83.1	82.8	83.7	83.4	82.8	82.8	82.8	81.3	86.2	86.2	86.2	86.8	86.5				**20**
21	83.1	82.8	83.1	83.7	83.1	83.1	83.1	82.8	83.7	83.4	82.8	82.8	82.8	81.3	86.2	86.2	86.2	86.8	86.5	100			21
22	84.4	84	84.4	84.4	82.8	82.8	82.8	82.8	83.4	83.1	81.6	81.6	81.6	80.1	85	85	85	85.6	85.3	97.8	97.8		22
